# Modeling and Optimization in Investigating Thermally Sprayed Ni-Based Self-Fluxing Alloy Coatings: A Review

**DOI:** 10.3390/ma13204584

**Published:** 2020-10-15

**Authors:** Katica Simunovic, Sara Havrlisan, Tomislav Saric, Djordje Vukelic

**Affiliations:** 1 Mechanical Engineering Faculty in Slavonski Brod, University of Slavonski Brod, 35000 Slavonski Brod, Croatia; shavrlisan@sfsb.hr (S.H.); tsaric@sfsb.hr (T.S.); 2 Faculty of Technical Sciences, University of Novi Sad, 21000 Novi Sad, Serbia; vukelic@uns.ac.rs

**Keywords:** Ni-based self-fluxing alloy coatings, designed experiment, regression modeling, optimization, statistical methods

## Abstract

In investigating thermally sprayed Ni-based self-fluxing alloy coatings, widely applied under conditions of wear, corrosion, and high temperatures, designed experiments and statistical methods as a basis for modeling and optimization have become an important tool in making valid and comparable conclusions. Therefore, this paper gives an overview of investigating Ni-based self-fluxing alloy coatings deposited by thermal spraying by the use of designed experiments and statistical methods. The investigation includes the period of the last two decades and covers the treatments of flame spraying, high-velocity oxy/air fuel spraying, plasma spraying, plasma-transferred arc welding, and laser cladding. The main aim was to separate input variables, as well as measured responses, and to point out the importance of correct application of statistical design of experiment. After the review of the papers, it was concluded that investigators have used the process knowledge to analyze and interpret the results of the statistical analysis of experimental data, which is in fact the best way of using the design of experiment in every research. Nevertheless, more attention should be given to correct planning and conducting the experiments to derive the models suitable for the prediction of measured response and which could be an appropriate input for single- or multi-objective optimization.

## 1. Introduction

Ni-based self-fluxing alloys were developed in 1950s as alloys of nickel with added Cr, B, Si and C (hence, the often-used terms NiCrBSi, NiCrSiB, NiCrSiBFe, Ni-Cr-B-Si, etc.). They are applied in conditions of wear, corrosion and high temperatures. Boron and silicon (1 to 5 wt.%) added to nickel improve the fluxing properties and act as deoxidizers, forming borosilicate protecting other elements from oxidation [1,2]. In addition, boron and silicon (so-called temperature suppressants [2]), as well as chromium (10 to 20 wt.%), lower the melting temperature of pure nickel [2]. With the addition of chromium, good corrosion resistance is achieved, and thanks to the addition of carbon (up to 1wt.%), carbides are created, which, along with borides, silicides, carboborides, and some other phases in the nickel matrix, increase the hardness (even up to 70 HRC, depending on the chemical composition of the coating material). Iron, molybdenum, tungsten, and different hard particles or rare earth elements can be added, as well. With application in almost all areas of human activity (power plants, engines, conditions of extreme wear and corrosion, aerospace industry, molds and tools, machine parts, chemical industry, automotive industry, food processing industry, etc.) [1,2,3], these alloys can be an alternative to hard chromium plating, which is not environmentally friendly due to the dangers of depositing chromic acid and carcinogenic effects on the respiratory organs of humans. Their application in nuclear plants was considered, due to the replacement of cobalt alloys, which are more expensive, and, in addition, cobalt enhances the effect of radiation.

The most frequent thermal spraying deposition technologies [1,2,3,4,5,6] used to deposit these alloys are plasma-transferred arc welding (PTAW or PTA welding), laser cladding (LC), flame spraying (FS), plasma spraying (PS), and high-velocity oxy/air fuel spraying (HVOF, HVAF), while less often applied are detonation gun spraying and electric arc spraying. All thermal spraying deposition technologies produce thick coatings (according to [1,2,6], from 30 micrometers to several millimeters; for PTAW, even 20 mm). PTAW is a welding coating (overlay) process, but also usually included in thermal spraying group [1]. It uses electric discharge to obtain the coating (mainly from powder) that is metallurgically bonded to the substrate and without porosity, i.e., with excellent density. Laser cladding is a very similar deposition technology, but uses the energy of laser to melt the powder or wire. With both of these technologies, the substrate melts and the coating material mixes with the substrate material (this is called dilution). In flame spraying, the thermal energy of fuel-oxygen/air flame is used to obtain molten particles of coating material (in the form of powder, wire, road, cord or liquid). Since the protective atmosphere is usually not provided, the forming of oxides in the coating can be very frequent. The velocities of a stream of molten particles are low (below 100 m/s) and coatings of high porosity (between 10% and 20%) are obtained. Nevertheless, this is the most widely used procedure due to its low cost. In plasma spraying, usually powders are melted in plasma (particle velocities are up to 200 m/s), thus forming the coating. To prevent oxidation, apart from atmospheric plasma spraying, the vacuum plasma spraying can be applied. In HVOF or HVAF process, the supersonic speeds are generated (up to 700 m/s) by the use of a Laval nozzle. Due to higher particle velocities, the porosity decreases and the adhesive (bond) strength increases. The melting (remelting, fusing or fusion) is frequently performed after the flame and plasma spraying, at temperatures up to 1100 °C (depending on the chemical composition of the Ni-based self-fluxing alloy) with the main aim to bond the molten particles in the coating (to remove lamellar splat structure) and thus reduce the porosity and increase the cohesive strength and additionally, to achieve metallurgical bond between the coating and substrate, i.e., to increase the adhesive (bond) strength (>80 MPa). The investigated alloys are originally developed as an alloying system (addition of boron and silicon and often chromium and nickel) for using in the sprayed and molten state to obtain coatings with less porosity and metallurgical bond between the coating and the substrate. The fusing process is frequently carried out after spraying (two-step process). The usual ways of subsequent remelting are by flame, laser, and in the furnace. However, in the investigations presented in Reference [7,8,9,10,11], NiCrBSi coatings were obtained by one-step process. In investigation Reference [9], the coatings were deposited by plasma spraying system with modified nozzle for enhancing particle heating with an internal powder injector. The used system enabled higher particle temperatures thus providing a self-fusing effect between molten droplet and previous splat, i.e., the additional post-spray fusing was avoided in that way.

Nevertheless, it has to be mentioned that, except for thermal spraying, Ni-based self-fluxing alloy coatings can also be obtained by sintering [12], casting, brazing, induction cladding [13], etc. In addition, there are various combinations of deposition technologies, for instance, in Reference [14,15], where the authors investigated vacuum brazing of NiCrBSi alloy with added WC-Co or WC-10Ni, respectively, previously obtained from flexible coated cloth technology developed in 1960s and 1970s.

Thermally sprayed Ni-based self-fluxing alloy coatings are exposed to different conditions in exploitation. That is why they are explored from nearly all aspects, i.e., investigations are aimed at those coatings’ resistance to different types of wear and to corrosion [3], but the influence of external mechanical loads, residual stresses, as well as microstructures, have also been investigated [16]. While investigating, many authors employed a scientific approach to designing the experiment.

For almost a hundred years now, the investigators from nearly all fields of science have been using a modern statistical approach in designed experiments in order to acquire new knowledge and draw conclusions about the investigated problem. The term “designed experiment” (experimental design, statistically designed experiment, statistical design and analysis of experiment, design of experiment—DOE) implies a scientific and systematic approach to planning of experimental investigation in order to conduct it efficiently, saving time and finances. Sir Ronald A. Fisher made a considerable contribution to this modern and systematic statistical approach to designing the experiments. In the twenties and early thirties of the last century, by working and discussing with many investigators, he concluded that only a well-designed and conducted experiment could provide valid conclusions. After such well-designed and conducted experiments, the obtained experimental data need to be statistically processed and analyzed and then, based on the results, the technological, i.e., process, conclusions reached. Such a systematic approach requires adhering to the following guidelines for designing the experiment [17]:

- Recognition of and statement of the problem (being a teamwork it includes people well acquainted with the investigated process, statisticians, operators, laboratory technicians, etc.).
- Selection of the response variable (what will be measured).
- Choice of input variables (factors), levels, and ranges (what will be varied).
- Choice of experimental design (as types of experimental designs are numerous, which one will be chosen depends on what the experiment is aimed at and which processing phase it is in).
- Performing the experiment (three important principles, described below, are to be taken into the consideration).
- Statistical analysis of the data (it is necessary to apply the knowledge of statistics).
- Conclusions and recommendations (“from statistics” turn again to the investigated problem and use the process knowledge to explain statistical conclusions).

In addition to these seven steps, it is also necessary to apply three basic principles in conducting an experiment, originated from the above-mentioned Fisher. These are randomization, replication, and blocking (more detailed in Reference [17,18]). However, it happens sometimes that investigators do not apply these principles in experimental investigations (or do not know that they have to apply them) and, accordingly, this can lead to wrong conclusions. This has also been noticed by the review of literature on the application of designed experiments and statistical methods in investigating thermally sprayed Ni-based self-fluxing alloy coatings.

Therefore, in the present paper, the authors made a review of investigations of widely applied thermally sprayed Ni-based self-fluxing alloy coatings, including the statistical approach, in order to systematize input variables (what has been varied) and responses (what has been measured). The aim is also to point out the importance of observance of the three previously mentioned principles (randomization, replication, blocking) in experimental design and generally the importance of the correct planning and conducting of experiments and statistical analysis. The structure of most thermally deposited coatings can be very unpredictable since it is obtained by high-velocity deposition and cooling processes. It can be even dependent on the dimensions of the sample [7]. Consequently, in investigating these coatings, it is especially good to have at least the replication principle fulfilled.

Furthermore, the present paper is a continuation of or addition to the research presented in authors’ two-part paper [3,16] as regards thermally sprayed Ni-based self-fluxing alloy coatings. The collected important information on Ni-based self-fluxing alloy coatings was presented in these papers in a systematic way, and the diversity of research, for the period from 2000 to 2013, was pointed out, by citing 360 references. In connection with DOE methodology, this paper can be an addition to the research described in Reference [19,20]. In investigation Reference [19], Pierlot et al. reviewed the design and analysis of experiments methodology application in thermal spraying by comparing different experimental designs and evaluating their application. The author of Reference [20] also showed the most important types of the applied designed experiments in the field of thermal spraying, from screening experiments to those applied in the optimization phase. He also considered the methods of artificial intelligence (neural networks and fuzzy logic), showing their advantages and disadvantages with regard to the methodology of designed experiments. Through the review of papers, the author also proved the combination of designed experiments and methods of artificial intelligence where the designed experiments were in fact used as a basis to obtain the data that were afterwards processed by the methods of artificial intelligence.

After the previous general insight into the application of Ni-based self-fluxing alloy coatings and statistical design and analysis of experiments, the rest of the article is organized as follows:

- In Section 2, the investigations in which the designed experiments are applied for obtaining regression models, reviewed, and presented.
- Section 3 presents the research in which investigators did not apply (or did not specify) designed experiments for obtaining regression models or used some statistical methods.
- The investigations in which the DOE methodology and methods of artificial intelligence are combined, or only DOE methodology is used with the main aim of optimization, are also covered in Section 4.
- In Section 5, the discussion on the certain models, methodology, or optimization is presented.
- Finally, the conclusions are provided in Section 6.

## 2. Statistical Modeling with DOE in Investigating Ni-Based Self-Fluxing Alloy Coatings Deposition

DOE methodology has important applications in the investigation of processes and products with the main aim of improving or optimizing their performance. In addition, it can serve as an important tool even in the development phase of a new process or product. Most authors have used the results of designed experiments for modeling, i.e., obtaining statistical (empirical, regression, mathematical) models for the prediction of response. For that purpose, researchers have frequently applied full factorial designs (factorials) where all levels of the factors are combined. Partial designs–fractions were used, as well, and some authors applied central composite design (CCD).

Section 2.1 to Section 2.4 present the review of the papers where statistical modeling approaches applying DOE were used in investigating Ni-based self-fluxing alloy coatings deposition technologies.

### 2.1. PTAW and Laser Cladding

Dependence of response (mass loss) of PTA welded Ni-based self-fluxing alloys coatings (thickness 4–5 mm) at dry sliding wear–pin on disc method, on coating hardness (which depends on subsequent heat treatment), testing temperature, and sliding distance was obtained in Reference [21], by the application of full factorial design with three factors at three levels. It was concluded that testing temperature was a significant factor.

In the paper of Ramachandran et al. [22], authors applied central composite design to study wear rate at dry sliding wear of Ni-based self-fluxing alloys (along with cobalt-base alloy and stainless steel) deposited by PTAW on carbon steel (in thickness of 3 mm). The yield and ultimate tensile strength of Ni-based self-fluxing alloy was lower (310 and 655 MPa, respectively) than that of cobalt-base and higher than that of stainless steel. This does not apply to the microhardness that was highest for Ni-based self-fluxing alloy (480 HV0.1). Three factors were being changed (hardness and revolution speed of rollers and normal load at pin on roller test), each one at five levels. By statistical analysis of data, regression models were obtained with high coefficient of determination for the coating wear rate and the three previously mentioned coatings. It was concluded that the rollers’ hardness was of considerable effect on response. Results and conclusions of the research, but only for Ni-based self-fluxing alloys, given in Reference [23], are similar to those in Reference [22]. Research of abrasive slurry of different PTA welded deposits of 3-mm thickness (Ni-based self-fluxing alloys, cobalt-base alloy, and stainless steel) was conducted in the paper by Ramachandran et al. [24]. Four different input variables were being varied at five levels (size of river sand particles in water, temperature and concentration of slurry, and number of revolutions of the samples), forming a central composite design of experiments with the aim to obtain statistical models of the wear rate dependence on the mentioned input variables. By increasing the size of river sand particles and the concentration of slurry (the highest influence), the wear rate was also increased, while, by increasing revolution speed of the samples, the wear rate was decreased. The slurry temperature variable had no considerable effect on the wear rate. Results and conclusions of research, but only for Ni-based self-fluxing alloys, given in Reference [25], are similar to those in the previously reviewed paper [24]. Ni-based self-fluxing alloy behaved different in sliding [22,23] and abrasive slurry [24,25] wear compared to cobalt- and iron-based alloy. This type of alloy had the lowest wear rate for sliding wear and the highest for abrasive slurry wear.

Reciprocating pin on plate sliding testing of 660-µm thick laser cladded and laser textured Ni-based self-fluxing alloy coatings was conducted by Garrido et al. [26]. They applied two sets of designed experiments. In the first experiment, at three levels, there were changes: the ratio between the surface covered by dimples and the whole texturing density and the geometric factor–the ratio between the diameter and the dimples depth (aspect ratio) aimed at defining the Stribeck curves. In the second experiment, instead of the first, previously mentioned factor, the distance between microdimples was defined at three levels, as well as combined with the microdimples diameter (as it was proved that the depth depends on the diameter) at three levels. The effect on the coefficient of friction change was observed with the aim to define the minimum one. It is demonstrated in this paper that experiments are an iterative process [17] in which the previously performed experiments are used to draw conclusions and design the next experiments.

Influence of three factors (laser power, powder feed rate, and scanning speed), changed at three levels, on the dimensions of Ni-based self-fluxing alloy coating (clad height, clad width, and clad penetration depth into substrate) was researched in paper by Davim et al. [27] with the aim to obtain a regression model. For the confirmation experiment, the authors compared actual and predicted values and calculated the error, which was acceptable for clad height and width (7.6% and 6%, respectively), while, for the penetration depth, it was high (20.1%).

For the needs of numerical modeling (by finite element method) and aimed at estimating and comparing the height and penetration of Ni-based self-fluxing alloy coating and heat affected zone, Felde et al. [28] conducted a full factorial design changing two factors (scanning speed and laser power) at four levels. They concluded that, in some cases, there is a difference between the coating estimated dimensions obtained by simulation (numerical modeling) and the dimensions of real samples because all possible parameters of influence on the complicated process of laser alloying for modeling were not considered.

Garrido et al. [29] studied laser texturing of laser cladded Ni-based self-fluxing alloy coatings (>660 µm thick, of 800 to 880 HV0.3 microhardness), by applying central composite design. They changed three input variables (energy, laser spot diameter, and impulse duration) with the main aim to obtain statistical dependence of the diameter and the dimples’ depth on the previously mentioned input parameters.

Laser single-pass and multi-pass cladding of NiCrBSi+WC coatings was investigated by Dubourg and St-Georges [30], with the aim of obtaining statistical dependence between laser cladding parameters (scanning speed, powder feed rate, overlapping, and out-of-focus distance) and width, height, and penetration depth of the laser clad, as well as the content of WC in the coating. The Taguchi experimental design was combined with the experimentation and modelization design of experiments. The second mentioned method is an interactive technique that enables a simultaneous varying of all factors. Scanning speed and powder feed rate were significant factors influencing the clad geometry.

### 2.2. Flame Spraying

**Figure 1:**
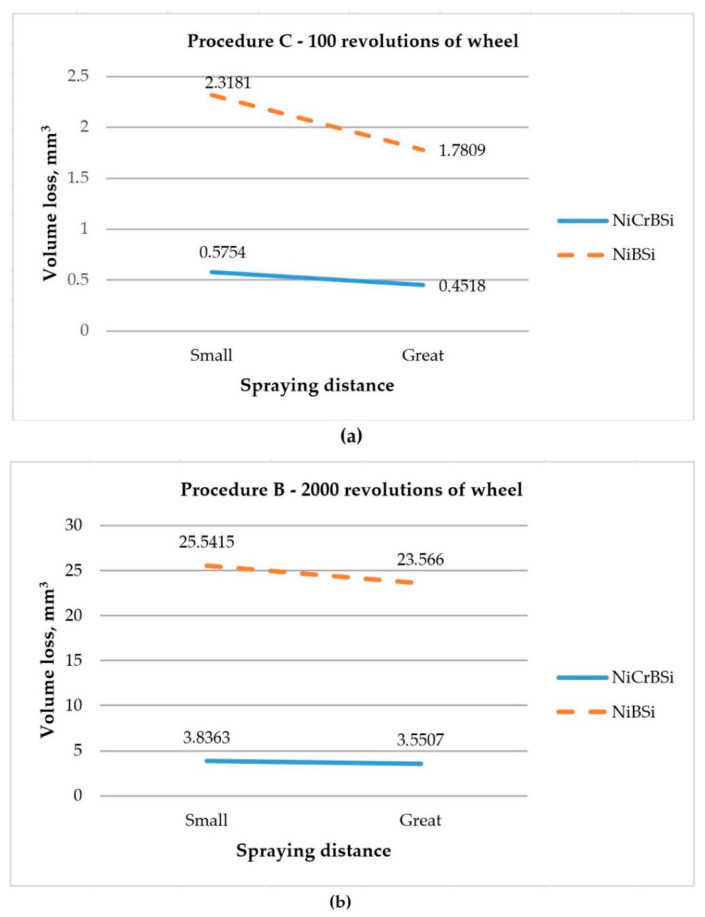
Interaction between spraying distance and type of coating: (**a**) Procedure C; (**b**) Procedure B [11].

Resistance to abrasive wear of flame sprayed and simultaneously fused Ni-based self-fluxing alloy coatings (NiCrBSi and NiBSi) of 1-mm thickness, with the application of dry sand/rubber wheel test, was investigated by Havrlisan et al. [10]. By applying full factorial design 2^2^ with three replicates, for two standardized variants (100 and 2000 revolutions of wheel), authors obtained the regression models, which showed dependence of volume loss on spraying distance and type of applied coating. For the shorter variant of testing, it was proven that the spraying distance, along with the type of coating, was significant, as well the interaction between these two factors (Figure 1a), while, for the longer variant, only the type of coating was significant (Figure 1b). Authors concluded that this might be connected with the microstructure in the coating surface and greater data variability for repetitions in longer variant and affected different coating layers with different structures. In that way, the authors of this paper (who are also the authors of the present investigation) explained and corroborated the statistical conclusions by process knowledge and thus complied with the last step in the guidelines for designing the experiment [17], shown in Section 1.

In their research of flame sprayed and furnace fused Ni-based self-fluxing alloy coatings, Bergant et al. [31] applied mixed-level factorial design varying the factor time of fusing at two levels and the factor temperature of fusing at three levels. The conclusion they reached was that the lowest porosity was at temperature 1080 °C and time of 10 min. While analyzing variance, it was concluded that the porosity was influenced by the temperature and time of subsequent heat treatment, as well as that interaction between factors was low. After the fusion process at 1080 °C for 20 min, the coating thickness decreased (from an average of 445 µm for a sprayed state to an average of 329 µm), due to the increase in density and entrapped gases release; in addition, the surface roughness *Ra* decreased from an average of 9.88 µm to an average of 3.26 µm). The lowest average porosity of 0.87% was obtained after furnace fusing at 1080 °C for 10 min.

Resistance to abrasive wear (pin on disc test) of flame sprayed Ni-based self-fluxing alloy coatings (of 0.95 to 1.05 mm thickness) with addition of 0.4% of CeO_2_ and 0.6% of La_2_O_3_ was analyzed while changing four factors (size of abrasive, loading, temperature, sliding speed) at three levels [32]. This author also calculated the difference between actual (measured) and predicted (obtained by the statistical models) values, i.e., error. In contrast to the usual amount of chromium (about 15%), a Ni-based self-fluxing alloy with 1.2 to 1.6% chromium was used here. The porosity decreased from 6.2 to 5.4% due to the addition of rare earth oxides. In addition, rare earth oxides influenced the increase of hardness (from 210 ± 9 HV5 to 241 ± 12 HV5) due to the refinement of structure and the higher amount of eutectic.

### 2.3. Plasma Spraying

Fernandez et al. [33] investigated the influence of four input variables at two levels—load, amount of hard WC particles in the coating, size of abrasive testing particles, and type of testing (dry and wet)—on the abrasion mass loss. The thickness of the coatings was 2–3 mm, and hardness of 855 ± 20 HV0.3 was obtained for coatings without WC, while, for the coatings with WC added, the hardness of the matrix was 850 ± 20 HV0.3, and, for the WC, it amounts to 1290 ± 20 HV0.3. It was proven that the size of abrasive testing particles and presence of hard particles in the plasma sprayed and remelted Ni-based self-fluxing alloy coating are of great influence to abrasive wear.

Valente [34] applied factorial design in investigation of controlled atmospheric plasma sprayed and induction remelted coatings of 300 µm thickness by varying the process parameters, power and spraying distance, at two levels, and the third factor, powder volumetric fraction (NiCrBSi and Mo powder), at three levels. The author concluded that the coating containing 10% Mo and 90% NiCrBSi powder had low porosity (0.9%), high hardness (668 HV0.3), good corrosion resistance, and good properties of wear.

Load, testing temperature, presence of WC in coating, and deposition technologies were varied at two levels in the investigation of Rodriguez et al. [35] when they applied reciprocating pin on plate sliding wear test for Ni-based self-fluxing alloy coatings of 300 to 500 µm thickness, plasma, and flame sprayed with remelting. The hardness of plasma sprayed coatings was 50 to 55 HRC, while for the flame sprayed and fused it was 60 HRC. After fusing, the surface roughness decreased to 2–4 µm, when comparing to flame sprayed state (20–30 µm). All factor levels combinations were tested, i.e., the full factorial design was applied with responses–the mass loss of coatings but also of counter body (alumina) after sliding wear testing. After statistical analysis of the wear data, it was concluded that the significant factors were the kind of applied thermal spraying and load. That the two other factors, temperature and WC particles, were not significant, was explained by the fact that Ni-based alloys were wear resistant at higher temperatures and that due to the bad connection between WC and the matrix the WC particles were pulled out of the matrix; thus, they did not contribute to the higher resistance to wear.

Tribological properties of plasma and HVOF sprayed Ni-based self-fluxing alloy coatings with solid lubricant Fe_2_O_3_ were studied by Zorawski and Skrzypek [36]. For plasma spraying, the input variables were the amount of Fe_2_O_3_, spraying distance, gas pressure, and current, while, for HVOF treatment, along with the amount of Fe_2_O_3_ and spraying distance, the flow rate of oxygen and propane was also changed. The responses were as follows: surface roughness, coefficient of friction, and microhardness. For the mentioned properties, for both spraying treatments, by statistical analysis of experimental data obtained by the fractional design of experiment, the authors developed linear regression models. Spraying distance and amount of Fe_2_O_3_ solid lubricant showed as significant factors of influence on surface roughness, but for the HVOF procedure only, while the amount of Fe_2_O_3_ solid lubricant was a significant factor for the coefficient of friction of the HVOF deposited coatings. For plasma sprayed coatings, the maximum microhardness was 642 HV0.5, and, for HVOF, sprayed coatings, it was 706 HV0.5. Regarding the surface roughness *Ra*, the minimum value was 0.42 µm for plasma spraying, and, for the HVOF procedure, the minimum surface roughness was 0.18 µm. A lower porosity was achieved for the HVOF process (1.13 ± 0.56%) than with the plasma spraying process (3.19 ± 1.76%).

### 2.4. HVOF Spraying

Resistance to pin on disc wear and corrosion of three types of HVOF sprayed coatings (NiCrBSi, WC-12%Co, and Cr_3_C_2_-25%NiCr) of 150 to 170 µm thickness was investigated by Shabana et al. [37]. For the pin on disc wear examination they performed mixed-level factorial design changing two factors (load and temperature) at two levels, while the third factor (sliding distance) was changed at five levels. Statistical models of the wear dependence on the mentioned factors were obtained with high coefficients of determination. When comparing these three types of coatings, NiCrBSi coatings had the lowest adhesive strength (58.15 MPa) and lowest microhardness (997 HV0.05), while the porosity was lower (amounts to 0.92%) than the porosity for Cr_3_C_2_-25%NiCr (1.49%).

Gisario et al. [38] examined laser remelting of HVOF deposited Ni-based self-fluxing alloy on aluminium alloy substrate. Mixed-level full factorial experiment was conducted changing two factors, laser power and rotational scan speed at six and five levels, respectively, with two replications of factor levels combinations. Effectiveness index of laser remelting was the response for which the nonlinear regression model was obtained, providing the conclusion that a better ratio will be achieved by higher laser power and smaller rotational speed. Effectiveness index was defined as the ratio of the thickness of the coating affected by laser remelting and the total thickness of the coating.

Analysis of resistance to abrasive wear (pin on disc test) of HVOF Ni-based self-fluxing alloy coatings with addition of WC and CeO_2_ was conducted in Reference [39]. Due to the addition of 0.4% CeO_2_, the microhardness was increased from 1053 ± 113 to 1185 ± 96 HV0.1. Pointing out that a number of authors use experimental “one-factor-at-a-time” strategy [17], the author changed four factors at three levels (size of abrasive, load, temperature, sliding distance), with the aim to obtain important factors to response, average mass loss, but also a statistical model of abrasive wear that shows the dependence of response on input variables. Verification experiments were also conducted so as to verify the model in which loading, size of abrasive, and sliding distance were significant factors, with the first order interaction between load and size of abrasive, load, and sliding distance and between size of abrasive and sliding distance.

In Table 1, the investigations described in Section 2.1 to Section 2.4 are listed according to deposition technology and applied experimental designs.

## 3. Statistical Modeling and Different Statistical Methods in Investigating Ni-Based Self-Fluxing Alloy Coatings Deposition

There are authors who did not apply designed experiment (or did not mention this fact or there are no experimental data to make conclusion about the type of experimental design). They used statistical analysis of data from unplanned or unspecified experiments (or measurements) to obtain regression models (sometimes curve fitting term was used) or used some statistical methods to process and analyze the data without obtaining a regression model (e.g., Weibull distribution, descriptive and interferential statistics parameters).

The papers with statistical modeling and application of different statistical methods in investigating Ni-based self-fluxing alloy coatings deposition technologies are described in Section 3.1 to Section 3.4.

### 3.1. PTAW and Laser Cladding

Flores et al. [40] investigated erosion-corrosion resistance of PTA welded Ni-based self-fluxing alloy coatings with added WC varying the sand content in slurry, as well as temperature and velocity of slurry. Statistical dependences of the total mass loss (*TML*) on the slurry velocity (*v*) was obtained with high coefficients of determination. For Ni-based self-fluxing alloy coating, the statistical dependence, for the temperature 65 °C, was as follows: *TLM* = 0.0036 **^.^** *v*^3.1222^ with the coefficient of determination of 0.9984.

Badisch et al. [41] studied the influence of microstructure (average distance between WC particles, average diameter of WC particles and volume fraction of WC particles, matrix hardness, and shape of hard particles) of composite Ni-based self-fluxing alloy coatings obtained by PTAW and laser cladding on continuous impact abrasion wear. It was shown that the matrix hardness, along with the average diameter of WC particles, average distance between WC particles, and volume fraction of WC particles, had a great influence on continuous impact abrasion wear rate, while the shape of hard particles in the coating was of no significance.

Block on ring tests of resistance to sliding wear were applied in Reference [42] on laser cladded NiCrBSi coatings deposited to grey cast iron substrate. These authors obtained a statistical linear model for evaluation of the wear mass loss dependence on volume removed (coefficient of determination was 0.9962). By the statistical linear model Fernandez et al. [42] also evaluated average wear rate in dependence on the product of normal load and sliding speed (coefficient of determination was 0.8638). These authors measured the microhardness by coating depth at three different locations. The average microhardness was 900 HV0.3, with a decrease in hardness at the substrate/coating interface, due to, as the authors claim, the diffusion of Fe from the substrate. The decrease in hardness was more pronounced at the edges of the sample.

In studying the resistance to sliding wear of laser cladded NiCrBSi+WC coatings of 500 HV1 matrix hardness, Garcia et al. [43] obtained the exponential statistical dependence (with *R*^2^ = 0.8503) of the wear track cross section on the fraction of WC hard particles for different speeds of sliding, showing that the increase of the quantity of hard particles in a coating results in the decrease of wear. However, these authors mention marginal fraction of 27% of hard particles (actual concentration), above which there was not any significant reduction of wear. The authors measured the actual concentration of WC particles in the coating relative to the concentration in the feeder.

Linear single and multiple regression models (with high coefficients of determination) of abrasive wear rate dependence on parameters connected with WC particles (average distance between WC particles, average diameter of WC particles, and volume fraction of WC particles) in dry sand/rubber wheel abrasive test were developed in a study on laser cladded Ni-based self-fluxing alloy coatings, by Polak et al. [44]. They proved that matrix hardness did not affect mass loss while average distance between WC particles was of greatest significance (different from the study in Reference [41], where the matrix hardness was a significant factor for continuous impact abrasion wear).

Coefficient of determination for statistical linear dependence of hardness of a Ni-based self-fluxing alloy coating cladded by laser on stainless steel on the amount of added hard WC particles was presented in Reference [45]. The microhardness of the matrix increased from 300 HV0.3 to 350 HV0.3 when a smaller amount (5%) of WC particles was added, and, if a larger amount (45%) of WC particle was added, then the microhardness of the matrix increased to 900 HV0.3. These authors have the impact of wolfram carbide particles on intensity–laser-induced breakdown spectroscopy (method for characterization of coatings) presented by linear regression model, with the coefficient of determination of 0.999.

In Reference [46], which presents the results of investigation of NiCrBSi coatings deposited by cladding with CW CO_2_ laser, for three different particle sizes of NiCrBSi powder (smaller than 20 μm, from 20 to 80 μm and from 80 to 100 μm), the response surfaces were obtained that show the dependence of powder feed rate (g/min) on the pressure of carrier gas–air (MPa) and on the gas flow (dm^3^/min). They concluded that the pressure and the air flow increase resulted in the increase of the powder feed rate, but not equally for all particle sizes of the powder. Therefore, to continue the research, they used the powder particles of size 20 to 80 μm while the input variables were the cladding distance (changed at three levels, 10, 12, and 14 mm) and the laser spot speed (changed at five levels, 40, 60, 80, 100, 120 mm/min), while the response were the geometrical features of single laser track—height and width. Based on the conducted experiments and obtained regression models it was concluded that the increase of cladding distance resulted in the decrease of width and the increase of height, while the increase of laser spot speed resulted in the decrease of width and height of clad. The authors [46] think that the temperature gradients are the key factors for this.

Sliding wear (pin on disc) and Knoop hardness of laser cladded NiCrBSi coatings exhibited to aging in salt fog was investigated and the influence of aging time on mechanical properties was presented in Reference [47]. The authors used Weibull statistical analysis to analyze the Knoop hardness data, and, consequently, Weibull modulus versus aging time was obtained. For the aging time up to 800 h, the structure of the coating was homogenous (without pitting or crevice corrosion), and the wear properties were excellent (higher Weibull moduli were obtained).

Applying graphical software and statistical analysis (descriptive statistics) in the investigation, Ma et al. [48] proved that after laser remelting of previously laser cladded NiCrBSi coating with 50% added WC particles, the median size of particles was reduced from 35.4 to 5.62 μm. After laser remelting, there was a decrease in microhardness by 50 HV0.1 compared to microhardness after laser cladding (which was 900 to 1050 HV0.1).

### 3.2. Flame Spraying

Similar to Reference [42], a block on ring tests of resistance to sliding wear was applied in Reference [49], but, for flame spraying and flame remelting (*R*^2^ was 0.9822), as well as for laser remelting (*R*^2^ was 0.9874) for the dependence of mass loss on volume removed. After flame and laser remelting, the microhardness of the coating was approximately the same and was 900 HV0.1, which was lower than for flame spraying condition (1100 HV0.1).

In the paper of Stanford and Jain [50], the dependence of wear volume on wear time at pin on disc sliding test for flame sprayed NiCrBSi alloy coating (final thickness of 0.26 mm was achieved after grinding) is shown by a linear regression model with a high coefficient of determination *R*^2^ (0.9968), while the dependence of coefficient of friction on time could not be presented by a regression model with high coefficient of determination due to dissipation of data. The amount of porosity was 2.1%, and the hardness was 789 HV.

Sliding test with a lubricant into which debris particles (alumina Al_2_O_3_) were added was conducted for investigating flame sprayed and flame remelted NiCrBSi coatings of 1.1 mm thickness, in Reference [51]. Instead of initial surface roughness, authors suggested applying actual surface roughness in expressions for obtaining the coefficient of friction (Stribeck curves). For investigation, they used three different sizes of Al_2_O_3_ particles (1.5, 32.5, and 45 μm) and five kinds of normal load of 3, 6, 9, 15, and 20 N, and, for each load, the sliding speed was also changed. Curve fitting was applied for obtaining statistical dependence and coefficient of determination for Stribeck curves (coefficient of friction dependence on so-called lubrication number parameter). Included into this parameter was the initial surface roughness, lubricant viscosity, sliding speed, and contact pressure. With the aim to include the actual surface roughness, they also obtained the fitted curves with excellent coefficient of determination (0.9902) for the surface roughness dependence on debris particle size, and they included this expression, instead of initial roughness, into the expression for obtaining the coefficient of friction’s dependence on such new lubrication number into which the actual surface roughness was included.

### 3.3. Plasma Spraying

Fernandes et al. [52] statistically processed data to obtain confidence intervals for specific wear rate of plasma sprayed Ni-based self-fluxing alloy coatings with addition of ceramic ZrO_2_ particles; besides, using regression model, linear dependence was shown between frictional force and loading so as to compute the coefficient of friction, which was proven to decrease with ceramic particles added. These authors investigated coatings applied using two premixed powders and the dual system of feeding.

Atmospheric plasma spraying of NiCrBSi coatings with two sizes of powder particles (50 to 75 μm and 75 to 100 μm) was investigated in Reference [53]. By applying powder with smaller particles, a greater coating thickness (598 ± 24 μm) was achieved than the thickness (432 ± 20 μm) obtained by applying powder with larger particles. This was explained by the fact that larger particles sometimes cannot be melted and weaker adhering can be achieved. In investigating electrochemical corrosion in 3.5 wt.% NaCl solution, statistical approach was applied for obtaining statistical models of the following electrochemical impedance spectrum (EIS) data: solution, film, and charge transfer resistance, capacity element, Warburg impedance, and constant phase element. Chi-square test was used to compare the actual (measured), and the predicted (calculated) data and good correlation was proven.

Porosity of plasma sprayed Ni-based self-fluxing alloy coatings was the subject of research in Reference [54]. The aim was to obtain correlation and dependency between linear (by length) and the corresponding spatial (by volume) dimensional parameters of pores in a coating. Author Das [54] points to the need to evaluate 3D parameters of pores based on the analysis of the sample’s cross section, where the pores are represented as circles, while, in reality, they are pores in which the pore diameter cannot be well represented by the diameter of the cross-section circle as it depends on the section plane. Analysis of linear and spatial parameters was performed on plasma samples but also on the plasma and fused (sintered, as the author states) samples. Based on the calculated spatial parameters of the pores defined by linear parameters obtained on the sample’s cross section, it was concluded that the sintered plasma coatings had 100 to 400 times higher quantity of pores, but the relative porosity was lower due to the much smaller diameters of pores. After the sintering procedure, the relative spatial porosity decreased from 21–34% to 2.06–3.07%, and the pore diameter decreased from 350 μm to 38.4 μm.

From the statistical point of view, porosity of plasma sprayed Ni-based self-fluxing alloy coatings was analyzed in Reference [55]. Authors first checked whether the porosity data were normally distributed or according to the Weibull distribution. Additional statistical analysis was conducted in accordance with the Weibull distribution, and it was proven that the porosity of coatings was reduced as the hydrogen flow rate was increased. Authors’ explanation for this was higher temperature and speed of molten particles, therefore better bonding between the molten particles themselves in the coating. They also mentioned that increase of the hydrogen flow rate resulted in reduction of the residual stresses among molten particles. The third reason they gave for reduced porosity was also the captured air in which quantity was reduced with increased hydrogen flow rate. Based on dispersion of the data on porosity for lower hydrogen flow rates, it was concluded that this was the result of inhomogeneity of structure (high pores unevenly distributed). Investigations presented in Reference [55] were continued with the application of the same statistical approach, and the results were presented in Reference [56]. The authors concluded that the porosity was increased at lower spraying power due to microcracks and unmelted particles. They also mentioned the limit power of 57 kW above which the quantity of cracks was not practically reduced any longer because all of the particles were melted already. Spraying power influence on the porosity but also mechanical properties (modulus of elasticity, microhardness, and residual stresses) was also investigated [57]. In Reference [58], the authors were varying powder feed rate and concluded that, at higher powder feed rates, there was quite a number of unmelted particles and inhomogeneity in the plasma sprayed coating leading to the increase of porosity, but the modulus of elasticity, microhardness, and residual stresses were reduced till some minimum value and then increased with increasing powder feed rate. In Reference [59], the authors gave the results of investigations of porosity, modulus of elasticity, and microhardness related to the plasma spraying parameters (power, powder feed rate, and hydrogen flow rate) by using the same statistical approach as in Reference [55,56,57,58].

Frequently applied statistical Weibull distribution function for analysis of life data was used in investigations of Zhang et al. [60] and Zhang et al. [61], who studied rolling contact fatigue of plasma sprayed and laser remelted NiCrBSi coatings. In Reference [60], authors conducted 13 experiments in order to obtain the Weibull plot that shows predicted failure probability for different fatigue life data.

### 3.4. HVOF Spraying

Microstructure, hardness, modulus of elasticity, yield strength, surface roughness, and surface residual stresses of HVOF sprayed Ni-based self-fluxing alloy coatings of 230 μm thickness were investigated depending on spraying distance, in Reference [62]. Authors concluded that the increase of spraying distance resulted in the increase of the number of unmelted particles by volume, increase of surface roughness (from 8 to 11 μm) and decrease of the modulus of elasticity, while the linear statistical model proved the dependence of surface roughness *Rz* on the volume fraction of unmelted particles. These authors also presented the statistical dependence of interplanar spacing on sin^2^ψ (XRD measurement), as well as the dependence of indentation load on penetration depth. The higher compressive residual stresses for smaller spraying distances were also proven.

For dry sand/rubber wheel abrasion test, Miranda and Ramalho [63] obtained linear regression models of abrasive wear dependence on volume fraction of hard particles (WC+Co) for HVOF and flame sprayed Ni-based self-fluxing alloy coatings (of 0.5 mm thickness) applicable for the 30 to 70% fraction of hard particles. HVOF coatings had lower porosity (0.5%) and higher adhesive strength than flame sprayed coatings. The only exception is the flame sprayed and remelted coating with 30% WC+Co, for which the porosity was also only 0.5%.

Similar to Reference [48], Rukhande and Rathod [64] used descriptive statistics to obtain important parameters (mean, standard deviation, and median) for NiCrSiBFe powder particles and their distribution. They applied laser diffraction method for analysis of powder particles. The porosity was 2.21% for HVOF coatings and 3.09% for plasma sprayed coatings.

The results of investigating correlation between the properties of in-flight particles (temperature, velocity, and size) and the properties of plasma, flame, and HVOF sprayed NiCrBSi coatings (amount of oxides, porosity, adhesive strength, hardness, and modulus of elasticity) are presented in Reference [65]. The authors obtained the same coating thickness of 300 μm for all three procedures. The authors concluded that for the HVOF deposition technology the best properties achieved due to highest velocities of in-flight particles, i.e., the highest kinetic energy of particles at impact resulting in strong cohesion and hardness of the coating. The statistical processing of the collected data was performed to obtain the average values and standard deviations. The velocity of particles has been shown to depend on their diameters. The larger the diameter, the lower the speed. This is most pronounced in the HVOF process, where velocities of 500 and 400 m/s were achieved for smaller and larger particles. In the plasma spraying procedure, the velocities were 160 and 120 m/s. For the flame spraying process, the velocities were the lowest (41 and 35 m/s) and did not differ significantly for larger and smaller particle diameters.

Those type of investigations reviewed in Section 3.1 to Section 3.4 are summarized in Table 2, following the classifying principle from Section 2.

## 4. Optimization in Investigating Ni-Based Self-Fluxing Alloy Coatings Deposition

To optimize a process or product design, response surface methodology (RSM) [17,18,19,20,66] and Taguchi approach [19,20,67,68] have often been using. Along with the DOE methodology, the artificial intelligence methods are also applied in thermal spraying among other methods for optimization, as shown in a review paper [20], previously cited.

In the following Sections, the papers with optimization approaches in investigating Ni-based self-fluxing alloy coatings deposition technologies are presented.

### 4.1. PTAW and Laser Cladding

In the research by Siva et al. [69] dealing with plasma transferred arc welding (PTAW) of Ni-based self-fluxing alloy to stainless steel, authors varied the travel speed, current, oscillation amplitude, preheat temperature and powder feed rate at five levels, aimed at predicting weld bead dimensions and dilution and at obtaining optimal parameters, by applying the combination of designed experiment (central composite design, CCD) and genetic algorithms, GA. In Introduction, these authors point out the precedence of application of genetic algorithms over other optimization methods, particularly the method of steepest ascent (descent) that is applied in response surface methodology (RSM) when reaching the region of optimal parameters. Optimal values of PTAW parameters for obtaining minimum depth of penetration to substrate, maximum height and width of weld bead and minimum dilution, obtained by the application of GA approach (minimum penetration 0.317 mm; maximum height 4.165 mm; maximum width 23.973; and minimum dilution 5.632%), proved better than the values obtained by Generalized Reduced Gradient (GRG) method (minimum penetration 0.36 mm; maximum height 4.115 mm; maximum width 23.973; and minimum dilution 6.356%), built into the Excel Solver and applied on non-linear models.

Tu et al. [70] aimed at finding optimal parameters of PTA welding for two types of coatings (Stellite cobalt alloy and Ni-based self-fluxing alloy) on 0.48% carbon steel. Two methods were applied and compared, the Taguchi method and the Taguchi regression method for obtaining optimal values of accelerating voltage, powder feed rate, and preheat temperature (these factors were identified as significant), as well as of current, waving oscillation, plasma gas rate, and rotation, which will give minimal sliding (pin on disc) wear mass loss. For both applied methods, Stellite was in optimal combination and the Taguchi regression method gave better signal to noise ratio than the Taguchi method. When comparing the actual and predicted values of mass loss, i.e., signal to noise ratios, for the Taguchi method, an average error of 7.05% was obtained, and for the Taguchi regression method, the error was 5.55%,

Three different responses (volume fraction of WC/W_2_C particles in the coating, hardness of the coating matrix, and equivalent diameter of WC/W_2_C particles) depending on three different input variables (parameters) of PTA welded Ni-based self-fluxing alloys were investigated by Ilo et al. [71] using the grey relational Taguchi method with the aim to obtain optimal parameters (welding current, welding speed and oscillation speed). The authors concluded that it is important to determine optimal parameters because of the considerable influence they have on the dissolution of carbides in Ni matrix. By reducing the volume fraction of WC/W_2_C particles in the matrix (because of dissolution), the matrix hardness will be increased, but, at the end, the resulting hardness will be decreased because of lower microhardness of new phases, which can be of influence to resistance to abrasive wear. Depending on the combination of process parameter levels, the minimum matrix microhardness was 518 HV0.1, while the maximum value was 785 HV0.1.

Laser cladding of Ni-based self-fluxing alloy on steel with 0.95% carbon was investigated by Onwubolu et al. [72] who applied the combination of factorial design and artificial intelligence (scatter search optimization as a method of evolutionary algorithms). The response was the clad angle (which must be large enough to avoid porosity, according to the authors), calculated by means of the clad width and height, input variables being scanning speed, laser power and powder feed rate, whose optimal values after application of scatter search algorithm were the following: scanning speed 5 mm/s, power 2 kW and powder feed rate 5 g/min.

Wu et al. [73] studied the porosity of laser cladded NiCrBSi coatings mainly with the aim of obtaining a statistical model and its application for optimization. Input variables were laser power, scanning speed and powder feed rate, each varied at three levels. Response was the porosity area that was set during optimization to be minimal. They have proven that the most significant factor was powder feed rate. With optimal parameters, laser power of 1524.80 W, scanning speed of 6.72 mm/s, and powder feed rate of 5.20 g/min, a porosity rate of 0.01% was achieved. Their study also proved that porosity could not be completely avoided by the application of the suggested optimal parameters, and concluded that the powder without porosity should also be used, as well as such a deposition process applied, which would not require a high shielding gas flow.

### 4.2. Flame Spraying

The application of Taguchi method for obtaining optimal values of substrate surface roughness and preheat temperature, oxygen-acetylene ratio (type of flame) and spraying distance is presented in References [74,75], aimed at obtaining maximum adhesive strength of flame sprayed and furnace remelted Ni-based self-fluxing alloy coatings on low carbon steel. Optimum values, verified by a new confirmation experiment, as well, were as follows: substrate material surface roughness 9.2 μm, substrate preheat temperature 200 °C, spraying distance 200 mm, and flame type–carburizing with obtained average adhesive strength of 22.35 MPa.

**Figure 2:**
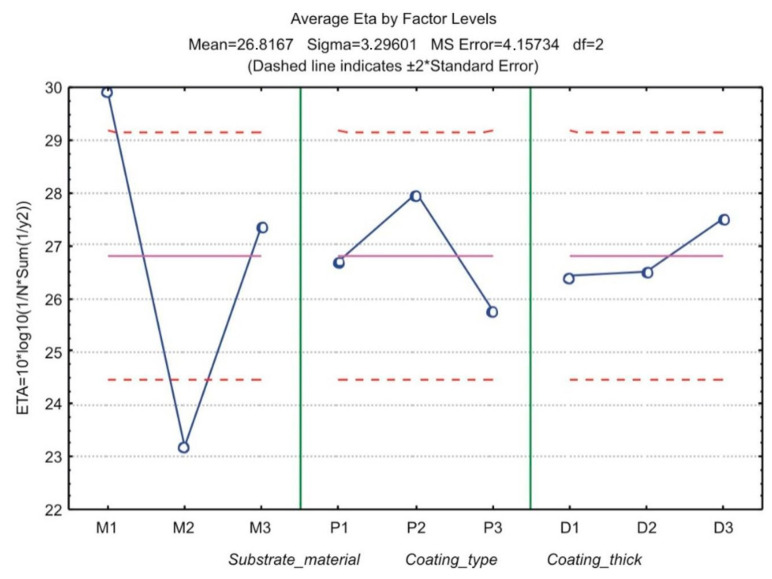
Common graphical presentation of S/N (signal-to-noise) ratios (ETA) after applying the Taguchi method [8].

By the use of Taguchi method, cracking resistance of three types of flame sprayed and simultaneously fused NiCrBSi coatings was investigated and the results are presented in Reference [8]. The author proved that NiCrBSi+WC coating (P2) of thickness 0.8 mm (D3) flame sprayed and simultaneously fused on steel C45 (M1) should be most cracking resistant in three-point bending test (Figure 2). In order to check the optimal combination, the author conducted a confirmation experiment for which an average critical force of 33 kN was obtained.

Figure 2 is a common presentation after applying the Taguchi method. Taguchi defined the evaluation method, which is S/N ratio (signal-to-noise ratio). The combination of the levels of control factors that has the highest S/N ratio is optimal, i.e., the construction is more robust, i.e., less sensitive to noise factors. In investigation Reference [8], the expected S/N ratio was 31.077 ± 1.88, and the calculated S/N ratio after the confirmation experiment was 30.35 and was in the expected range of 29.19 to 32.95.

**Figure 3:**
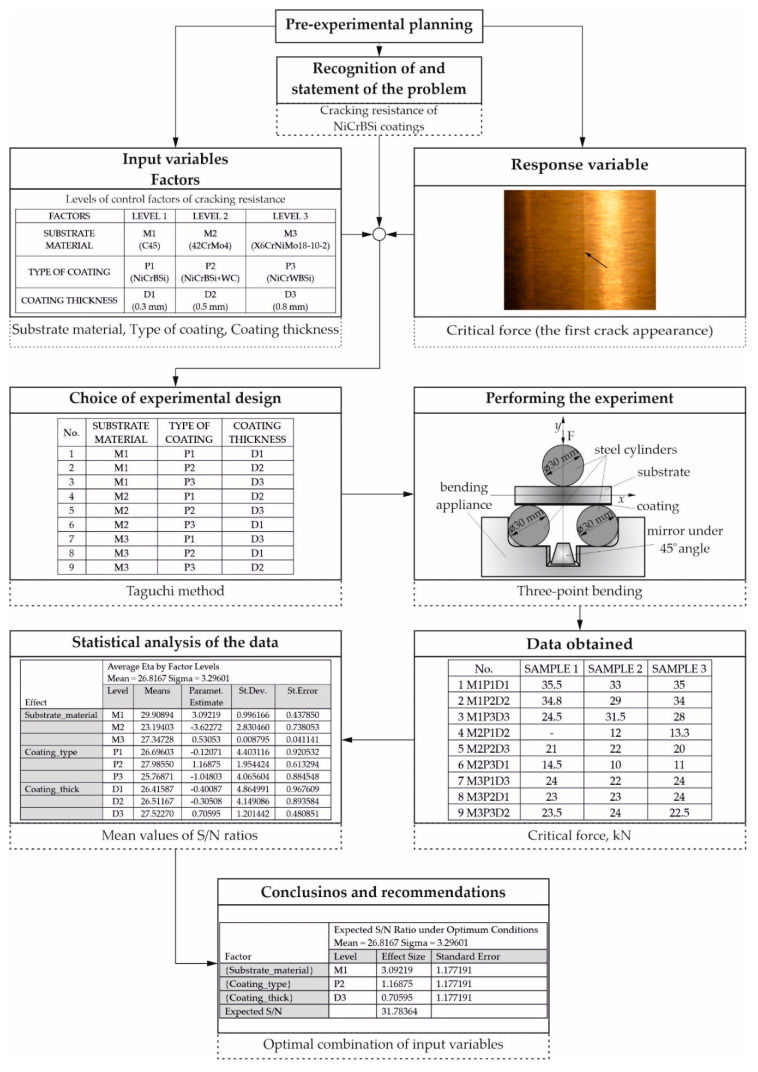
Adhering to the guidelines for designing the experiment [8].

The guidelines for designing the experiment listed in Section 1 of the present paper were followed in Reference [8], which is presented in Figure 3.

### 4.3. Plasma Spraying

Two factors, percentage of laser remelted surface and the angle of laser meshing were changed at four levels, in the investigation of Vijande et al. [76], who studied plasma sprayed and partially laser remelted Ni-based self-fluxing alloy coatings. The result of performed full factorial design was a linear regression model with a high coefficient of determination, which showed wear mass loss dependence on the two previously mentioned factors. Then, the input variables optimal values (50% of laser remelted surface and the angle of meshing of 22.5°) were calculated to obtain minimal mass loss at lubricated wear.

Liu et al. [77] optimized the parameters (powder feed rate, power, argon and hydrogen flow rate) for supersonic plasma spraying, applying the Box-Behnken designed experiment. Response was the NiCrBSi coating porosity. Optimal values for input variables were the following: 90 g/min (powder feed rate), 50 kW (power), 3.1 m^3^/h (flow rate of argon), and 0.35 m^3^/h (flow rate of hydrogen), and estimated porosity was 1.8%.

In addition to the powder feed rate, power, and plasma gas flow rate, Dubrovskaya et al. [78] optimized some more plasma spraying parameters (spraying distance, rotational speed of the specimen, and the displacement of the spraying torch) by the application of the steepest descent method in investigating Ni-based self-fluxing alloys with the addition of zirconium and hafnium. The responses were coating microhardness, porosity and thickness, the presence of cracks, and adhesive strength. The authors calculated the optimal values of the input variables, which were as follows: spraying distance 100 mm, rotational speed of the specimen 20 rpm, displacement of the spraying torch 10 mm/min, powder feed rate 40 g/min, power 36 kW, and plasma gas flow rate 50 l/min.

### 4.4. HVOF Spraying

Rukhande and Rathod [64] applied the Taguchi method to investigate the influence of the type of deposition technology (HVOF and plasma spraying), hardness of counter body (alumina, silicon nitride, and bearing steel) and normal load (10, 15, and 20 N) at dry sliding test on wear mass loss and coefficient of friction (responses). They proved that the hardness of counter body exerted the most significant influence on responses, followed by the type of deposition technology while normal load exerted the least influence. The smallest load (10 N), with the smallest hardness of the counter body (bearing steel) gave the lowest mass loss for the HVOF procedure.

Full factorial design was performed by Gil and Staia [79] while investigating corrosion resistance and porosity of HVOF sprayed Ni-based self-fluxing alloy coatings, varying spraying distance, powder feed rate, and fuel/oxygen ratio at three levels, while repeating all combinations of factor levels two times. They showed that all three factors exerted a significant influence on the result—corrosion current density and corrosion potential in 3.5% NaCl solution, but that only the spraying distance was of influence on porosity (for smaller distances, the porosity was even lower due to higher speed and higher temperature of melted particles). Optimal parameters were also defined: powder feed rate 60 g/min, spraying distance 380 mm, and fuel/oxygen ratio from 1.1 to 1.2.

Taguchi experimental design was applied for investigating HVOF and HVOLF (L–liquid) NiCrBSi alloy coatings deposited on the steel substrate [80]. Four factors were varied in slurry erosion examination: speed, concentration of slurry, impact angle, and size of abrasive particles. It was proven that the last-mentioned factor exerted no significant impact on wear rate.

Table 3 summarizes the previously reviewed studies of Ni-based self-fluxing alloys, in which some optimization method was applied.

## 5. Discussion

**Figure 4:**
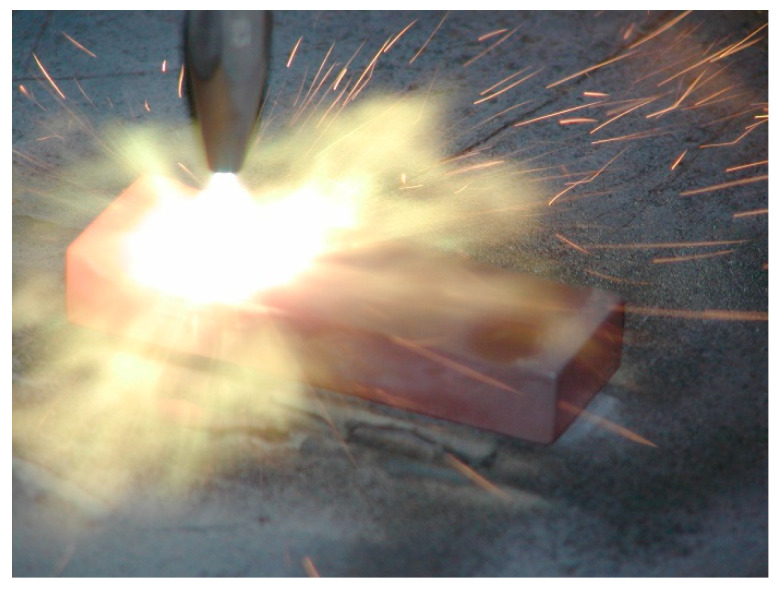
Flame spraying with simultaneous fusing—one-step process.

After reviewing the papers, it was noticed that, in addition to the statistical part, the microstructure of coatings was examined in most of the investigations. However, more attention should have been paid to the influence of deposition technology (or fusing) on the structure of the substrate. Evidence of a change in structure of coating, coating/substrate interface, and substrate is given due to high fusing temperature, depending on the type of substrate (and related thermal properties), coating thickness, but also on dimensions of the sample, which is proven in one of the previous works [7] of the authors of this paper. Figure 4 presents the flame spraying and simultaneous fusing, from which it is evident that there is a significant influence of heat on the substrate.

It has already been stated that the fusing process is frequently performed after the flame and plasma spraying, at temperatures up to 1100 °C. However, some authors, especially for flame spraying, do not specify the method of fusing or do not mention it at all. In addition,, sometimes neither the type of material nor the dimensions of the substrate are stated.

The authors very often do not even state the number of samples. Due to the nature of the thermal spraying technology (high cooling rates), the structure of the coatings is often unpredictable and it is good to have the replication principle fulfilled. In addition to this principle, the investigators should adhere to the other two principles of experimental design, which are randomization and blocking [17,18]. Therefore, the authors of this paper would like to suggest to the investigators to use more samples for the same combination of the levels of input variables. Likewise, randomization should be considered, as well as blocking, if necessary.

**Figure 5:**
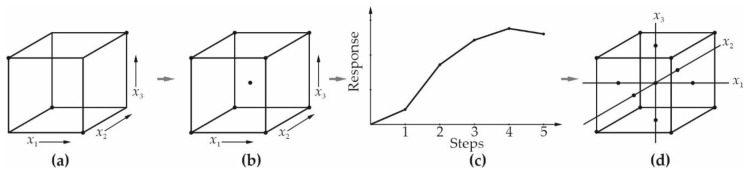
Scheme of response surface methodology (RSM) possible steps: (**a**) Fractional factorial; (**b**) full factorial with added centre points; (**c**) method of steepest ascent; (**d**) central composite design (CCD); adapted from Reference [17].

It was concluded that all authors have used engineering or process knowledge to analyze and interpret the results of the statistical analysis of experimental data, which is the single and best way to use DOE in any research. However, it has to be specified which design or designs of experiment were applied and why those types were chosen. For instance, given that RSM is a methodology which consists of several steps (Figure 5) and is intended for optimization, researchers should distinguish between the terms RSM and response surface. The latter means the statistical model (i.e., the dependence of response on input variables) represented graphically by the surface.

**Figure 6:**
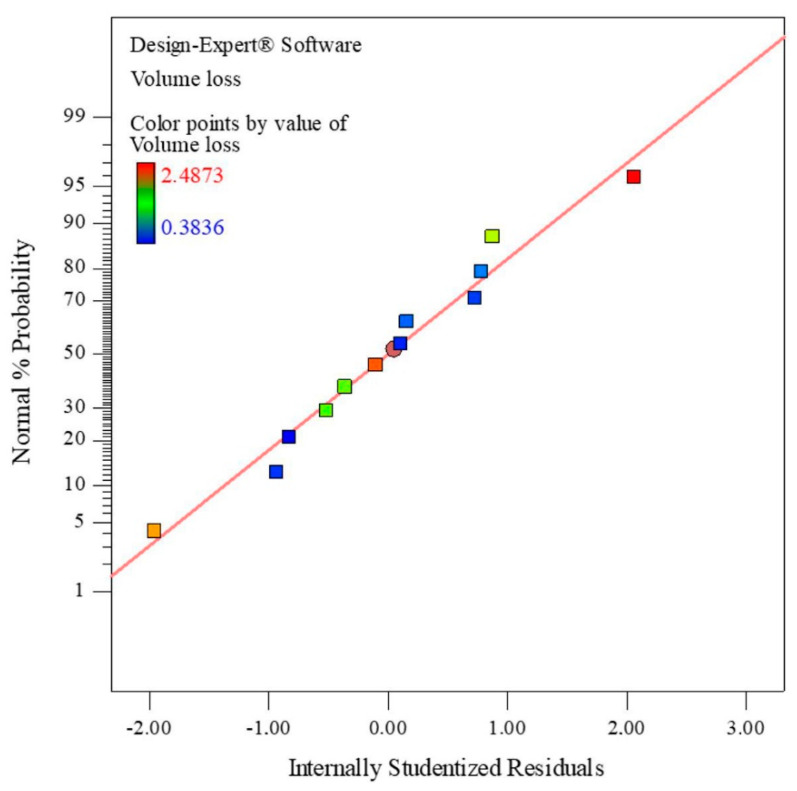
Normal probability plot of internally studentized residuals.

When it is about regression models, the authors of the present investigation would like to comment on this topic. By statistical processing of experimental data, using some statistical software, it is not enough just to derive a regression model. It needs to be checked, especially if used as input to single- and multi-objective optimization. In addition to analysis of variance (ANOVA) of the model, the diagnostics should include the following:

- Normal probability plots of the residuals (or internally studentized residuals) to check for normal distribution (Figure 6).
- Plots of internally studentized residuals versus predicted values to check for constant error.
- Detailed discussion of externally studentized residuals to look for outliers, i.e., influential values (analysis of measure difference in fits and indicator of Cook’s distance should be included).
- Checking the coefficients of determination (high and equivalent values of *R*^2^ adjusted and *R*^2^ for prediction are desirable), as well as other ANOVA output (standard deviation, i.e., MSE—mean square error, predicted residual sum of squares, coefficient of variation, adequate precision, etc.).

Moreover, significant interactions between factors of the experiment, as well as significance of factors, must be interpreted from the point of view of process knowledge. The possibility of model transformation must be considered, as well.

Giving the experimental data in table form is much better than only graphical presentation of obtained results or not even presenting the data at all. The regression model diagnostics or important model statistics should exist, and the software used for statistical processing and analysis should be mentioned.

## 6. Conclusions

Due to the fact that Ni-based self-fluxing alloy coatings are often applied in protection from wear, a number of authors investigated wear. For the analysis of resistance to wear (sliding, abrasion, erosion, impact abrasion, erosion-corrosion), the response in most cases was the mass or volume loss or wear rate or coefficient of friction and the input variables represented a combination of the spraying parameters and the testing parameters (load, temperature, sliding distance or speed, size of abrasive testing particles, environment, samples’ speed of revolution, impact angle, slurry concentration in investigation of slurry erosion, hardness, and number of revolutions of counter body) but also the properties of coatings (chemical composition of powders, presence of hard or solid lubricant particles, coating hardness, coatings with or without chrome added). Influence of the matrix hardness on wear was also studied.

In addition to wear, statistical approach was applied in the study of corrosion, adhesive strength, hardness, cracking resistance, surface roughness, and residual stresses. In treatments in which the effect on substrate is considerable (laser cladding, PTAW, and processes of remelting), geometric characteristics of the coating or heat-affected zone dependent on the treatment’s parameters were a frequent response. Designed experiments or statistical methods are applied in the study of porosity and rolling contact fatigue, as well.

In addition to frequently varying the spraying parameters dependent on applied deposition technology, as well as powder feed rate and preheat temperature of the substrate, the spraying distance has also been often varied. Some authors defined optimal spraying distance and powder feed rate and it was proven that importance of these factors can depend also on type of response or testing and deposition technology used. For research connected with laser cladding or laser remelting, mostly, all authors varied laser power and scanning speed.

Based on the given review of the application of designed experiments and statistical methods in investigating thermally sprayed Ni-based self-fluxing alloy coatings, it can be concluded that a certain smaller number of authors combined designed experiments with artificial intelligence methods mainly aimed at obtaining optimal parameters of the applied deposition technology. It is obvious that there is an additional opportunity for research here. A paper was even found in which a combination of numerical modeling and experimental research was conducted, in which the results obtained by numerical modeling were compared with experimental values obtained by the application of designed experiment. Here, too, the possibility for further research exists. The designed experiments have also been applied with the aim to obtain optimal values of input variables for reaching minimal or maximal values of response but also to obtain regression models for demonstrating the dependence of response on input variables. In the research of some authors, designed experiments were not applied or specified, but the research results were demonstrated by statistical models, or some of the statistical methods were applied.

The authors believe that the topic of this investigation can be useful in experimental research of any type of thermally sprayed coatings. It can also be useful in research in which statistical methods and designed experiments are applied.

## Figures and Tables

**Table 1:** Summary of investigations for the modeling purpose.

Deposition Technology	Input Variables	Responses	Method	References
PTAW	Coating hardness; Testing temperature; Sliding distance	Mass loss (dry sliding wear–pin on disc)	Full factorial design 3^3^	[21]
	Hardness of roller; Revolution speed of roller; Normal load	Wear rate (dry sliding wear–pin on roller)	CCD	[22,23]
	Size of river sand particles in water; Temperature and concentration of slurry; Number of revolutions of samples	Wear rate (abrasive slurry)		[24,25]
LC	Distance between microdimples obtained by laser texturing; Microdimples diameter	Coefficient of friction	Full factorial design 3^2^	[26]
	Laser power; Powder feed rate; Scanning speed	Clad height, width and penetration depth into substrate	Full factorial design 3^3^	[27]
	Laser power; Scanning speed	Height and penetration of coating; Heat affected zone	Full factorial design 4^2^	[28]
	Energy; Laser spot diameter; Impulse duration	Microdimples’ diameter and depth	CCD	[29]
	Scanning speed; Powder feed rate; Overlapping and out-of-focus distance	Clad height, width and penetration depth into substrate; Content of WC in the coating	Taguchi and experimentation and modelization DOE	[30]
FS	Spraying distance; Type of coating	Volume loss (three-body abrasive wear)	Full factorial design 2^2^	[10]
	Time of fusing; Temperature of fusing	Porosity area; Corrosion resistance	Mixed-level full factorial design	[31]
	Size of abrasive; Load; Temperature; Sliding speed	Mass loss (pin on disc two-body abrasive wear)	Fractional factorial design	[32]
PS	Load; Amount of WC particles in the coating; Size of abrasive particles; Environment (dry and wet testing)	Mass loss (three-body abrasive wear)	Full factorial design 2^4^	[33]
	Power; Spraying distance; Powders volume fraction	Porosity; Fraction of unmelted particles; Microhardness; Dynamic coefficient of friction and mass loss (dry sliding wear–ball on disc); Slurry erosion number; Polarization resistance	Mixed-level full factorial design	[34]
PS FS	Load; Testing temperature; Presence of WC in powder; Deposition technologies	Mass loss of coating and counter body (dry sliding wear–pin on plate)	Full factorial design	[35]
PS HVOF	Amount of Fe_2_O_3_ in Ni matrix; Spraying distance; Gas pressure; Current–PS Amount of Fe_2_O_3_ in Ni matrix; Spraying distance; Flow rate of oxygen and propane–HVOF	Surface roughness; Coefficient of friction (dry sliding wear–ball on disc); Microhardness	Fractional design	[36]
HVOF	Load; Temperature; Sliding distance	Mass loss (dry sliding wear–pin on disc)	Mixed-level full factorial design	[37]
	Laser power; Rotational scan speed–Laser remelting	Effectiveness index for laser remelting	Mixed-level full factorial design	[38]
	Size of abrasive; Load; Temperature; Sliding distance	Mass loss (pin on disc two-body abrasive wear)	Fractional factorial design	[39]

PTAW—plasma-transferred arc welding; LC—laser cladding; FS—flame spraying; PS—plasma spraying; HVOF—high-velocity oxy fuel spraying.

**Table 2:** Summary of investigations without or not specified designed experiments.

Deposition Technology	Input Variables	Responses	Method	Reference
PTAW	Content of sand; Slurry temperature; Velocity of slurry	Mass loss (erosion-corrosion)	Curve fitting	[40]
PTAW LC	Average distance between WC particles; Average diameter of WC particles; Volume fraction of WC particles; Matrix hardness; Shape of WC particles	Wear rate (continuous impact abrasive wear)	Regression model	[41]
LC	Volume removed (worn); Load; Sliding speed	Mass loss (dry sliding wear–block on ring); Average wear rate	Curve fitting	[42]
	Weight % of WC	Wear track cross section (dry sliding wear–ball on disc)	Curve fitting	[43]
	Average distance between WC particles; Average diameter of WC particles; Volume fraction of WC particles; Matrix hardness	Wear rate (three-body abrasive wear)	Regression model	[44]
	Weight % of WC	Hardness; Intensity (laser-induced breakdown spectroscopy)	Curve fitting	[45]
	Pressure of carrier gas (air), Flow of carrier gas (air); Cladding distance, Laser spot speed	Powder feed rate; Clad height and width	Regression model	[46]
	Exposure time in salt fog	Weibull modulus (Knoop hardness distribution)	Weibull distribution	[47]
	-	-	Descriptive statistics	[48]
FS	Volume removed (worn)	Mass loss (dry sliding wear–block on ring)	Curve fitting	[49]
	Wear time	Wear volume (dry sliding wear–pin on disc)	Curve fitting	[50]
	Debris particle (Al_2_O_3_) size; Initial (or final) surface roughness, lubricant viscosity, sliding speed, contact pressure	Surface roughness; Coefficient of friction	Curve fitting	[51]
PS	Load	Frictional force (dry sliding reciprocating wear)	Confidence interval; Curve fitting	[52]
	Capacitance arc radius (Nyquist plot); Frequency (Bode impedance and phase plots)	Solution, film and charge transfer resistance; Capacity element; Warburg impedance; Constant phase element	Curve fitting; Chi-square test	[53]
	Linear parameters of pores	Spatial (3D) parameters of pores	Regression model; Correlation analysis	[54]
	Hydrogen flow rate	Porosity	Weibull distribution	[55]
	Power	Porosity	Weibull distribution	[56]
	Power	Porosity; Modulus of elasticity; Microhardness; Residual stress	Weibull distribution	[57]
	Powder feed rate	Porosity; Modulus of elasticity; Microhardness; Residual stress	Weibull distribution	[58]
	Hydrogen flow rate; Power; Powder feed rate	Porosity; Modulus of elasticity; Microhardness	Weibull distribution	[59]
	Contact pressure; Number of fatigue cycles	Failure probability	Weibull distribution	[60,61]
HVOF	Volume fraction of unmelted particles; sin^2^ψ (XRD measurement); Penetration depth	Surface roughness *Rz*; Interplanar spacing; Indentation load	Curve fitting	[62]
HVOF FS	Volume fraction of hard particles	Abrasive wear resistance (three-body abrasive wear)	Curve fitting	[63]
	-	-	Descriptive statistics	[64]
HVOF FS PS	-	-	Statistical distribution	[65]

PTAW—plasma-transferred arc welding; LC—laser cladding; FS—flame spraying; PS—plasma spraying; HVOF—high-velocity oxy fuel spraying.

**Table 3:** Summary of investigations for the optimization purpose.

Deposition Technology	Input Variables	Responses	Method	References
PTAW	Current; Oscillation amplitude; Travel speed; Preheat temperature; Powder feed rate	Weld height, depth and width; Dilution	Central composite design and genetic algorithm	[69]
	Type of coating material; Accelerating voltage; Powder feed rate; Preheat temperature; Current; Waving oscillation; Plasma gas rate; Rotation	Mass loss (dry sliding wear–pin on disc)	Taguchi and Taguchi regression method	[70]
	Current; Welding speed; Oscillation speed	WC/W_2_C particles volume fraction in the coating; Hardness of the coating matrix; Equivalent diameter of WC/W_2_C particles	Grey relational Taguchi method	[71]
LC	Laser power; Scanning speed; Powder feed rate	Clad angle calculated by clad width and height	Full factorial design 3^3^ and scatter search	[72]
	Laser power; Scanning speed; Powder feed rate	Porosity area	Method of steepest descent (RSM)	[73]
FS	Substrate surface roughness; Substrate preheat temperature; Oxygen-acetylene ratio; Spraying distance	Adhesive strength	Taguchi method	[74,75]
	Type of coating; Coating thickness; Type of substrate	Critical force (the appearance of the first crack)	Taguchi method	[8]
PS	Percentage of laser remelted surface; Angle of laser meshing for partial laser remelting after plasma spraying	Mass loss (lubricated sliding wear–block on ring)	Full factorial design 4^2^	[76]
	Powder feed rate; Power; Argon and hydrogen flow rate	Porosity	Box-Behnken design (RSM)	[77]
	Powder feed rate; Power; Plasma gas flow rate; Spraying distance; Rotational speed of the specimen; Displacement rate of the plasma torch; Alloying additive	Coating microhardness, porosity and thickness; Cracks; Adhesive strength	Method of steepest descent (RSM)	[78]
PS HVOF	Type of deposition technology; Hardness of the counter body; Load of the sliding wear test	Mass loss (dry sliding wear–ball on disc); Coefficient of friction	Taguchi method	[64]
HVOF	Spraying distance; Powder feed rate; Fuel/oxygen ratio	Corrosion current density; Corrosion potential; Porosity	Full factorial design 3^3^	[79]
HVOF HVOLF	Speed; Impact angle; Concentration of slurry; Size of abrasive particles	Mass loss (slurry erosion)	Taguchi method	[80]

PTAW—plasma-transferred arc welding; LC—laser cladding; FS—flame spraying; PS—plasma spraying; HVOF—high-velocity oxy fuel spraying; HVOLF—high-velocity oxy liquid fuel spraying.
